# Editorial: The expanding network of p53 signaling: Reaching to the unknown of cancer

**DOI:** 10.3389/fcell.2022.978056

**Published:** 2022-10-14

**Authors:** Xiang Zhou, Ji Hoon Jung, Hua Lu

**Affiliations:** ^1^ Fudan University Shanghai Cancer Center and Institutes of Biomedical Sciences, Fudan University, Shanghai, China; ^2^ Department of Oncology, Shanghai Medical College, Fudan University, Shanghai, China; ^3^ College of Korean Medicine, Kyung Hee University, Seoul, South Korea; ^4^ Department of Biochemistry & Molecular Biology and Tulane Cancer Center, Tulane University School of Medicine, New Orleans, LA, United States

**Keywords:** p53, metabolism, tumor microenvionment, cancer vaccine, cancer therapy

Four decades of p53 research since the discovery of this tumor suppressor have demonstrated that p53 prevents tumorigenesis by maintaining genomic stability and eliminates cancer cells by inducing cell growth arrest, necrosis, apoptosis, and ferroptosis (Levine and Oren, 2009; Levine, 2020). p53 activity is tightly controlled by various mechanisms. One master controller is the E3 ubiquitin ligase MDM2 that is encoded by a p53 target gene and inhibits the anti-tumor functions of p53 by alleviating its protein translation, blocking its transcriptional activity, and promoting its ubiquitination and proteolytic degradation (Lu, 2017; Zhou et al., 2017). Overcoming this negative feedback inhibition is crucial for p53 activation in response to different stress signals, including oncogenic stress, DNA damage stress, nucleolar stress, and nutrient restrictions (Zhou et al., 2015; Hafner et al., 2019). Also, p53 can be inhibited by other negative modulators, and thus, inactivating these modulators can lead to p53 activation as well (Zhou et al., 2017; Hafner et al., 2019). However, there are still numerous remaining questions in the p53 field. Is the canonical MDM2-p53 feedback circuit a really druggable target for the development of new anti-cancer therapies? What more could we learn about “gain of functions” (GOFs) of p53 missense mutants? Are there more molecular insights into the role of p53 in the maintenance of metabolic homeostasis and the prevention of cancer-induced metabolic remodeling? How do wild-type (wt) and mutant (mt) p53s regulate the immune response, and would generating mt p53 vaccine an effective and feasible approach for developing anti-cancer therapy? These outstanding questions are partly addressed in several nicely written review articles and novel research studies that are collected in this specific issue as briefly introduced below.

Blocking the MDM2-p53 feedback loop has been considered as a promising strategy to treat cancers harboring wt p53 for decades, although it has been quite challenging as there is not an applicable drug targeting this loop as an anti-cancer therapy in clinic use. In the issue, a review article by Kung et al. gracefully described a canonical mechanism by which oncogenic stress induces p53 activation with some new information and thoughts. Specifically, the oncogenic c-MYC or RAS signaling induces the expression of ARF, an alternate open reading frame encoded by CDKN2A, which in turn activates p53 by interacting with and inhibiting MDM2 activity. Importantly, they also summarized several potential therapeutic strategies targeting the ARF-MDM2-p53 cascade, including small molecules, peptides, and the proteolysis targeting chimera (PROTAC) tactic. In a research study, Han et al. reported that the PARP inhibitor olaparib, a targeted therapy for cancers with BRCA1/2 mutations or homologous recombination deficiencies, can induce p53 activation *via* RPL5/RPL11-mediated inhibition of MDM2 by triggering nucleolar stress, demonstrating an additional action mode of PARP inhibitors by targeting the nucleoli and activating the p53 pathway.

The p53-encoding gene, *TP53*, is the most frequently mutated gene in human cancers. The cancer-derived mutations of p53 include missense, frameshift, truncation, and deletion. Most of the p53 mutants are missense mutations that often occur in the DNA-binding domain of the p53 protein. These mutants not only lose their tumor inhibitory activity, but also exert a “dominant-negative” effect on the functions of wt p53. Remarkably, several hotspot mutants, such as mt p53-R175H, G245S, R248W/Q, R249S, R273H/C, and R282W, acquire GOFs to further promote tumor growth *via* diverse mechanisms (Freed-Pastor and Prives, 2012). Although these mutants usually lack the DNA-binding ability, they can indirectly regulate gene transcription by either binding to other transcription factors or modulating epigenetic modifications. Also, they can regulate other cellular processes through protein-protein interactions (Sabapathy and Lane, 2018; Zhou et al., 2019). As described in a review by Madrigal et al. in this issue, mt p53 regulation of microRNA expression involves both transcription-dependent and -independent mechanisms. Mt p53 was recently found to associate with replicating chromatin and PARP1 to facilitate aberrant DNA repair (Xiao et al., 2020). An interesting study by Annor et al. in this issue demonstrated that oligomerization of mt p53-R273H is not required for its chromatin association, though oligomerization of wt p53 is indispensable for its tumor suppressive activity. In a prospective essay by von Grabowiecki et al., the authors proposed a provocative idea that mt p53 might promote endosomal trafficking of a plethora of proteins involved in tumorigenesis and cancer progression by regulating Rab11-FIP1, which is supported by some recent studies as cited in this article and will await further validation.

Over the past years, growing evidence has revealed the crucial role of p53 in the maintenance of metabolic homeostasis and the prevention of cancer-associated metabolic remodeling (Labuschagne et al., 2018; Liu et al., 2019). Mammalian target of rapamycin (mTOR), an evolutionarily conserved serine/threonine protein kinase, serves as a central regulator by linking cellular nutrient status to cell growth. In this issue, Cui et al. offered a comprehensive review on the progresses of recent studies on the coordinated regulation of p53 and mTOR pathways in response to the physiological and genotoxic conditions. This is further consolidated by another review by Nagpal and Yuan, who elegantly collected numerous previous and new findings on the role of basally expressed p53 in restraining anabolic metabolism to prevent fast cell proliferation under non-stress conditions. In accordance, the tumor suppressive function of p53 has been also attributed to its activity to regulate glucose metabolism, lipid metabolism, amino acid metabolism, and iron metabolism in cancer cells, which are nicely illustrated in a review by Yu et al. Moreover, p53 has been shown to be involved in the regulation of recycling and clearance of metabolites, nutrients, and cellular debris. This line of information on wt and mt p53’s new functions is systematically reviewed by Rahman et al. They offered another comprehensive and updated view on the roles of wt and mtp53s in the regulation of autophagy signaling and provided new insights into the therapeutic potential by modulating p53-mediated autophagy.

The roles of wt and mt p53s in the regulation of inflammatory and immune responses have been a hot topic recently. To update this area of research, this issue has also collected several review and research articles. For instance, the review article by Nagpal and Yuan described that basally expressed p53 is required for the maintenance of immune homeostasis. In addition, Shi and Jiang offered a detailed review on various mechanisms underlying wt and mt p53 regulation of inflammation and immunity. For example, p53 prevents inflammation-associated cancer development by suppressing NF-κB and STAT3 signaling pathways (Gudkov and Komarova, 2016; Wormann et al., 2016), while mtp53 antagonizes the STING/TBK1/IRF3 pathway, resulting in tumor evasion of immune surveillance (Ghosh et al., 2021). Moreover, a research article by Zhang et al. showed that p53 mutation is associated the increased production of chemokines, leading to infiltration of different immunocytes in breast cancer. This suggests a complex tumor microenvironment in mt p53-harboring cancers. The development of vaccines targeting p53 has been an old, yet unsolved, topic, as both wt and mtp53 epitopes can be presented on the cell surface for T cell recognition (Houbiers et al., 1993). To update this interesting research area, Zhou et al. offered a thoughtful review on the recent progresses of vaccination of p53 or its peptides and discussed the possibility and application of p53-targeting vaccines to cancer treatment. Collectively, these review and research articles as published in this issue not only show recent progresses in various regulations and roles of wt and mt p53 in cancer development, progression, and immunology, but also provide more new insights into the p53 anti-cancer functions and mt p53’s oncogenic activities. Importantly, these articles also offer new thoughts and suggestions for targeting mt p53 as anti-cancer therapies, such as new potential approaches for developing p53 vaccines.

Although the canonical functions of p53 as a key regulator of cell cycle, DNA repair, and apoptosis have been well documented, increasing studies have been continuingly unveiling novel roles of p53 in metabolic remodeling, immune surveillance, and cancer therapy, making this magic molecule as the most attractive research target as well as a promising therapeutic target for developing anti-cancer therapies in the future. We are deeply grateful to these authors who have made great efforts to our better understanding of new functions of p53 and new insights into this still mysterious molecule by contributing their comprehensive review and elegantly-designed research articles to this special issue.
